# An Inventory of CiaR-Dependent Small Regulatory RNAs in *Streptococci*

**DOI:** 10.3389/fmicb.2021.669396

**Published:** 2021-05-25

**Authors:** Nancy Jabbour, Marie-Frédérique Lartigue

**Affiliations:** ^1^Université de Tours, INRAE, ISP, Tours, France; ^2^Centre Hospitalier Universitaire de Tours, Service de Bactériologie, Virologie, et Hygiène Hospitalière, Tours, France

**Keywords:** *Streptococci*, regulation, CiaRH, regulatory RNAs, csRNAs

## Abstract

Bacteria adapt to the different environments encountered by rapid and tightly controlled regulations involving complex networks. A first line of control is transcriptional with regulators such as two-component systems (TCSs) that respond to physical and chemical perturbations. It is followed by posttranscriptional regulations in which small regulatory RNAs (sRNAs) may affect RNA translation. *Streptococci* are opportunistic pathogens for humans and farm animals. The TCS CiaRH is highly conserved among this genus and crucial in bacterial survival under stressful conditions. In several streptococcal species, some sRNAs belong to the CiaRH regulon and are called csRNAs for *cia*-dependent sRNAs. In this review, we start by focusing on the *Streptococcus* species harboring a CiaRH TCS. Then the role of CiaRH in streptococcal pathogenesis is discussed in the context of recent studies. Finally, we give an overview of csRNAs and their functions in *Streptococci* with a focus on their importance in bacterial adaptation and virulence.

## Introduction

Due to their importance in the regulation of gene expression, small non-coding regulatory RNAs (sRNAs) are present in all kingdoms of life. The sRNAs were discovered in prokaryotes long before the first short interfering RNAs (siRNAs) and microRNAs (miRNAs) in eukaryotes. Adaptation to the environment involves a complex regulatory network in which sRNAs play an essential role. A decade ago, the high number of sRNAs discovered in various bacterial species was surprising (Brantl, 2009). Interestingly, these sRNAs differ in length, structure, and mode of action (Gottesman and Storz, 2011). However, sRNAs, 50–500 nucleotides long molecules, are often involved in the regulation of several cellular pathways and allow bacteria to adapt and survive under stressful conditions. All sRNAs are classified in several groups according to their location in the genome and their modes of action (Storz et al., 2011). In 1984, the first chromosomally encoded sRNA was discovered in *Escherichia coli:* MicF. This sRNA inhibits the translation of OmpF messenger RNA (mRNA) encoding the major membrane porin, OmpF (Mizuno et al., 1984). To respond to environmental changes, bacteria must first sense these changes, and two-component regulatory systems (TCS) are known to perform this function (Stock et al., 2000).

Streptococcal species infect humans and farm animals. Although some of them are commensal, other are responsible for severe infections in humans (Krzyściak et al., 2013). In *Streptococci*, many TCSs have been found. The TCS CiaRH was identified to be involved in natural competence and general virulence (Patenge et al., 2013). It is widespread among *Streptococci* but not found in another bacterial genus. Interestingly, it controls the expression of sRNAs called *cia*-dependent sRNAs (csRNAs) (Halfmann et al., 2007). This review concerns csRNAs identified in *streptococci*. It starts by highlighting the most important streptococcal species harboring a CiaRH and then analyzes the CiaRH TCS roles. Finally, this review focuses on all csRNAs identified until now and their functions.

## *Streptococcus* Species Harboring a TCS CiaRH

The *Streptococcus* genus is composed of chain-forming gram-positive bacteria including a large number of species (>100). Although this genus includes beneficial species such as *Streptococcus thermophilus*, used in the food industry for the production of yogurt (Blomqvist et al., 2006), streptococci are opportunistic pathogens, often involved in severe diseases in humans and farm animals. The major species in human infections are *Streptococcus pneumoniae, S. pyogenes*, and *S. agalactiae* (Krzyściak et al., 2013). *S. pneumoniae* is the main cause of community-acquired pneumonia, meningitis, and acute otitis media. *S. pyogenes* (Group A *Streptococcus*), an exclusively human pathogen, is involved in mild (pharyngitis, skin infections) to severe fatal invasive infections, such as necrotizing fasciitis and streptococcal toxic shock syndrome. Groups C and G *Streptococci*, such as *Streptococcus dysgalactiae* subsp. *equisimilis* and *Streptococcus equi*, are microbiologically similar to *S. pyogenes* (Baracco, 2019). *S. agalactiae* (Group B *Streptococcus*), a commensal bacterium of the gastrointestinal and genitourinary tracts, is the leading cause of neonatal infections, causing pneumonia, bacteremia, and meningitis via maternal transmission. As *S. pneumoniae*, *Streptococcus mutans*, *Streptococcus sanguinis*, *Streptococcus gordonii*, *Streptococcus mitis*, *Streptococcus oralis*, and *Streptococcus infantis* belong to the physiological flora in the human oral cavity. *S. mutans* is an opportunistic commensal species responsible for biofilm formation causing dental caries but also infective endocarditis. Conversely, *S. gordonii* and *S. sanguinis* are non-cariogenic colonizers. *Streptococcus gallolyticus* (and less frequently *Streptococcus lutetiensis*), an opportunistic bacterium inhabiting the gastrointestinal tract, is one of the main causes of infective endocarditis and is strongly associated with colorectal cancer (Pasquereau-Kotula et al., 2018). Among *Streptococci*, including *S. agalactiae* and *S. equi* mentioned above, some species can also infect animals, as *Streptococcus suis*, responsible for severe invasive, and often lethal diseases in swine and humans and *Streptococcus uberis*, main agent of mastitis in dairy cows (Keane, 2019). These bacteria must colonize, invade, and persist in the host. But above all, they must adapt to environmental changes and the various types of stress they encounter. One of the mechanisms that bacteria use to adapt and survive is the regulation of gene expression through the sRNA-mediated two-component regulatory systems.

## CiaRH: A Streptococcal Two-Component Regulatory System

**FIGURE 1:**
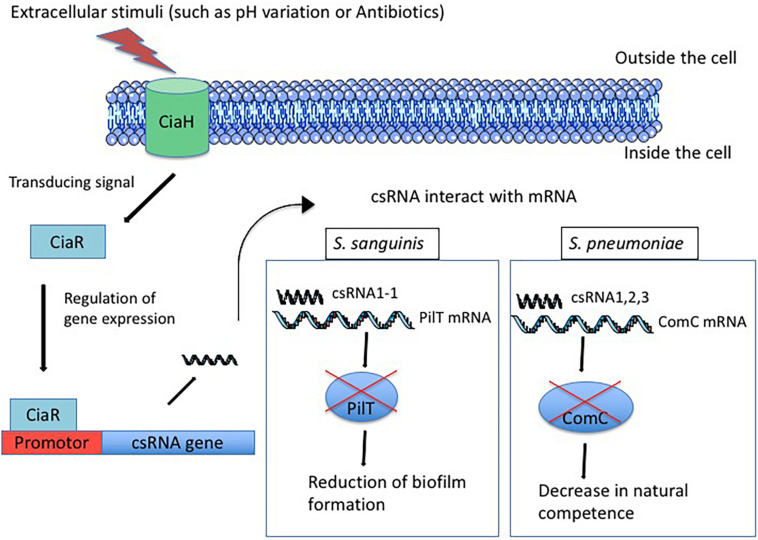
Regulation of csRNAs expression by TCS CiaRH after exposure of the cell to extracellular stimuli. First, the sensor CiaH detects extracellular stimuli such as pH variation and antibiotics exposure. CiaH transduces the signal to the regulator CiaR that interacts with promotor and allows csRNA expression. Then, csRNA interacts with target mRNA and allows or represses the translation. For example, in *S. sanguinis*, the csRNA1-1 interacts with the PilT mRNA and represses the translation of PilT protein inducing the reduction of biofilm formation. In *S. pneumoniae*, csRNA1 combined with csRNA2 and csRNA3 allows the repression of ComC translation, which reduces the natural competence of bacteria.

TCS CiaRH was first identified in *S. pneumoniae* while selecting for cefotaxime resistance in spontaneous laboratory mutants. CiaH is a histidine protein kinase anchored in the cytoplasmic membrane that receives information from the environment. It transmits the information to CiaR, a cytoplasmic response regulator that translates the signal into a cellular response by regulating the expression of targeted genes (Figure 1; Guenzi et al., 1994). The amino-acid sequence identity of CiaH and CiaR from different species ranges between 48–71 and 77–85%, respectively (Riani et al., 2007). In several species, CiaRH is involved in biofilm formation. In fact, the presence of SpeA (streptococcal pyogenic exotoxin A) in *S. pyogenes* leads to down-regulation of CiaRH expression genes and attenuates the biofilm-forming capacity, suggesting a link between TCS expression and biofilm formation (Babbar et al., 2019). In *S. sanguinis*, the deletion of the *ciaR* gene up-regulates the expression of arginine biosynthesis genes resulting in the formation of a fragile biofilm (Zhu et al., 2017). In *S. gordonii*, the inactivation of SdbA, a thiol-disulfide oxidoreductase, up-regulates CiaRH, which in turn leads to enhanced biofilm formation (Davey et al., 2016a). In *S. mutans*, the inactivation of CiaH gene affects the dental biofilm formation, abolishes bacteriocin production and competence development, suggesting the involvement of CiaRH in these phenotypes (Qi et al., 2004). Actually, the up-regulation of CiaRH in *sdbA* mutant *S. gordonii* strain leads to bacteriocin expression shutdown whereas inactivation of CiaRH restores bacteriocin production. Involvement of the TCS in bacteriocin expression indicates its importance in bacterial competition in order to colonize the host (Davey et al., 2016b). CiaRH is also known to influence streptococcal stress tolerance. TCS is involved in tolerance to acid and thermal stress in *S. mutans* (Liu and Burne, 2009b). In *S. gordonii*, mutation of the TCS leads a greater susceptibility of the mutant to low pH and oxidative stress than the wild type (Liu and Burne, 2009a). Moreover, CiaRH is involved in resistance to the immune system, intracellular survival, and virulence. Actually, in CiaR-deficient *S. agalactiae* strains, resistance to the immune system and intracellular survival are affected (Quach et al., 2009; Mu et al., 2016). The deletion of CiaRH in *S. suis* enhances the bactericidal activity of macrophages and attenuates bacterial virulence in animal models (Li et al., 2011; Zaccaria et al., 2016). Furthermore, the transcription level of the TCS is significantly higher in virulent than in strains of low virulence (Dong et al., 2015). As for *S. pneumoniae*, the CiaRH system prevents autolysis triggered by different conditions and allows the maintenance of a stationary growth phase (Mascher et al., 2006). In *S. pyogenes*, a *ciaH* mutant strain binds more efficiently to HEp-2 epithelial cells than the wild type. The internalization rate of the mutant is increased within the same range (Riani et al., 2007). Conversely, the deletion of CiaRH in *S. suis* exhibits a decrease in adherence to HEp-2 epithelial cells (Li et al., 2011). These conflicting results could be explained in two different ways. First, CiaRH-mediated regulation can be different between streptococcal species and under different conditions. Second, inactivation of only one gene of the TCS (*ciaH*) does not allow for obtaining the same phenotype as when both are inactive. In fact, when *ciaH* is inactive, *ciaR* may respond to other regulators whereas when only *ciaR* is inactive, *ciaH* may regulate other sensors. To summarize, CiaRH is involved in many cellular processes, including β-lactam resistance, lytic processes, biofilm formation, bacteriocin production, natural competence, virulence, and resistance to the immune system (Dagkessamanskaia et al., 2004; Sebert et al., 2005; Quach et al., 2009; Li et al., 2011). In *S. pneumoniae*, a direct repeat sequence, TTTAAG-N5-TTTAAG, has been identified by *in vitro* and *in vivo* transcriptional mapping as essential for the binding of CiaR. Fifteen promoters are controlled by CiaR, five of them (the strongest) drive the expression of sRNAs, which are designated csRNAs for *cia*-dependent sRNAs (Halfmann et al., 2007).

## Inventory of Csrnas Identified So Far

The sRNAs are classified into four major groups: CRISPRs (clustered regulatory interspaced, short palindromic repeats), riboswitches, protein-binding RNAs, and *trans-*acting RNAs. And the csRNAs belong to the *trans*-acting class and more precisely to the *trans*-encoded subclass. *Trans*-encoded RNAs are often expressed by genes located at a different locus from their target genes and thus share only a short and imperfect complementarity sequence with their target mRNAs. This imperfect complementarity allows *trans-*encoded RNAs to control more than one target mRNA and, therefore, to be part of complex regulatory networks. *Trans*-encoded RNAs, by forming a base association, affect the translation or the stability of the mRNAs. The interaction between an sRNA and its target mRNA leads to the inhibition of protein translation by blocking the ribosome binding site (RBS) or by inducing the degradation of the sRNA-mRNA duplex by RNases (Hui et al., 2014). *Trans*-encoded RNAs can also prevent mRNA degradation or activate mRNA translation by preventing the formation of an inhibitory structure that hides the RBS (Prévost et al., 2007). The FasX sRNA of *S. pyogenes* allows bacterial dissemination through two different mechanisms: first, by increasing ska mRNA stability, allowing the overexpression of streptokinase, and second, by hiding the RBS and decreasing pilus mRNA translation (Ramirez-Peña et al., 2010; Danger et al., 2015).

The five csRNAs, discovered in *S. pneumoniae* and validated by Northern blot analyses, are between 87 and 151 nucleotides long and have a high degree of similarity to each other. The presence of sequences complementary to RBS in these csRNAs indicates that they may control the initiation of translation. csRNA1 was shown to be a negative regulator of competence development (Tsui et al., 2010). The deletion of csRNA4 and csRNA5 revealed their role in autolysis control (Halfmann et al., 2007), and a mutant of csRNA5 was defective in a lung infection (Mann et al., 2012). The targets of these csRNAs were investigated by computational predictions with targetRNA and IntaRNA (Tjaden et al., 2006; Busch et al., 2008). Thirty-three predicted genes were tested by translational fusion, and six of them are possibly regulated by *S. pneumoniae* csRNAs (Schnorpfeil et al., 2013). The *spr0081, spr0371, spr0551*, and *spr1097* genes encode membrane-spanning proteins that belong to different transporter families. The *spr0159* gene encodes a protein harboring a DNA-binding domain and therefore is most likely to be a transcriptional regulator. The last one, *spr2043* (*ComC*), encodes the competence-stimulating peptide precursor (CSP), suggesting a link between CiaRH and competence control, mediated by csRNAs (Figure 1; Håvarstein et al., 1995; Schnorpfeil et al., 2013). It has been shown that each of the csRNAs down-regulates the *comC* gene, but they are not as effective alone as they are all together (Schnorpfeil et al., 2013). However, the combination of three csRNAs, csRNA1, 2, 3, or csRNA1, 2, 4, is sufficient to decrease the competence of *S. pneumoniae* (Laux et al., 2015). Interestingly, duplicated csRNA was observed in Hungarian *S. pneumoniae* serotype 19A isolate. Indeed, an internal sequence duplication is the cause of the carriage and expression of longer version of csRNA5 (Brantl and Brückner, 2014).

In the study of Marx et al. (2010), the presence of the CiaR binding site located in the intergenic regions and followed by transcriptional terminator was investigated in 14 streptococcal genomes. Thus, 61 candidate genes potentially express csRNAs. Among them, four were predicted in all *S. agalactiae* strains: *csRNA10, csRNA11, csRNA12*, and *csRNA13*. Their expression was confirmed in NEM316 by RNA sequencing and the first three were also validated by Northern blot. The genes were renamed *srn015*, *srn024*, *srn070*, and *srn085*, respectively. The corresponding csRNAs were overexpressed at low pH (5.2), suggesting they could contribute to acid stress resistance (Rosinski-Chupin et al., 2015). However, no function and no targets have been assigned to them yet.

In *S. sanguinis* SK36, six csRNAs were predicted (csRNA1-1, csRNA1-2, csRNA1-3, csRNA2, csRNA7, and csRNA8) and confirmed by Northern blot (Marx et al., 2010). Target prediction and a luciferase reporter assay allowed the identification of the *pilT* gene, a constituent of the type IV pilus gene cluster, to be the target of *S. sanguinis* csRNA1-1. The interaction between csRNA1-1 and the *pilT* mRNA was proved by RNA-RNA electrophoretic mobility shift assay (EMSA). Furthermore, csRNA1-1 and csRNA1-2 arranged in tandem in *S. sanguinis* genome are probably duplicated genes that negatively regulate biofilm formation (Ota et al., 2017; Figure 1). This observation suggests the implication of csRNAs in the host colonization by this species and provides further evidence concerning the involvement of csRNAs in bacterial adaptation.

The csRNA genes in *S. pyogenes* MGAS315 (csRNA14, csRNA15, and csRNA25) were also predicted, and the expression of csRNA15 and csRNA25 was confirmed by RNA sequencing (Marx et al., 2010; Le Rhun et al., 2016).

Five csRNAs in *S. mitis* B6 (csRNA1, csRNA2, csRNA3, csRNA4, and csRNA5) and five other csRNAs in *S. oralis* Uo5 (csRNA1, csRNA2, csRNA3, csRNA4, and csRNA6) were predicted and confirmed by Northern blot (Marx et al., 2010).

Other csRNAs have been predicted in *S. mutans* UA159, *S. suis* 05ZYH33, *S. gordonii*, *S. gallolyticus*, *S. dysgalactiae*, *S. equi*, *S. uberis*, and *S. thermophilus* but not confirmed so far (Marx et al., 2010; Table 1 and Supplementary Table 1). The identification of csRNAs of new viridans streptococci obtained from primates indicates that all csRNAs predicted previously in *S. mitis*, *S. gallolyticus*, *S. gordonii*, and *S. oralis* are present in the new strains studied. Two species with unknown csRNAs contain csRNAs from other species. Indeed, *S. infantis* harbors four of the five *S. oralis* Uo5 csRNAs, and *S. lutetiensis* harbors the *S. gallolyticus* UCN34 csRNAs except for csRNA40 (Denapaite et al., 2016).

**TABLE 1:** csRNAs predicted in streptococcal species.

**Species**	**Strain**	**csRNA**	**Screening method**	**Confirmed by**	**Length (nt)**	**Direct target**	**Mechanism of action**	**Regulatory function**	**References**
*S. pneumoniae*	R6 D39	csRNA1	PredictRegulon server	Northern blot	93	*comC*, *spr0081*, *spr0159*, *brnQ*, and *spr1097*	Translational repression by base pairing	Autolysis and competence modulation	Halfmann et al., 2007; Marx et al., 2010; Tsui et al., 2010; Schnorpfeil et al., 2013
		csRNA2			96				
		csRNA3			98				
		csRNA4			92				
		csRNA4			148				
		csRNA5							

*S. agalactiae*	NEM316	csRNA10	Non-coding RNA gene finder	RNA-seq, Northern blot	145	ND	ND	ND	Marx et al., 2010; Pichon et al., 2012; Rosinski-Chupin et al., 2015
		csRNA11			96				
		csRNA12			118				
		csRNA13			218				

*S. mitis*	B6	csRNA1	Non-coding RNA gene finder	Northern blot	94	ND	ND	ND	Marx et al., 2010
		csRNA2			97				
		csRNA3			99				
		csRNA4			96				
		csRNA5			146				

*S. mitis*	SF100	csRNA2	Non-coding	NC	98	ND	ND	ND	Marx et al., 2010
		csRNA6	RNA gene finder		200				

*S. oralis*	Uo5	csRNA1	Non-coding RNA gene finder	Northern blot	95	ND	ND	ND	Marx et al., 2010
		csRNA2			98				
		csRNA3			100				
		csRNA4			93				
		csRNA6			200				

*S. sanguinis*	SK36	csRNA1-1	Non-coding RNA gene finder	Northern blot	89	*pilT*	Putative translational repression by base pairing	Inhibition of biofilm formation	Marx et al., 2010; Ota et al., 2017
		csRNA1-2	Non-coding		93	ND	ND	ND	Marx et al., 2010
		csRNA1-3	RNA gene	Northern blot	87				
		csRNA2	finder		96				
		csRNA7			85				
		csRNA8			176				

*S. pyogenes*	MGAS315	csRNA14	Non-coding	RNA seq	68	ND	ND	ND	Marx et al., 2010;
		csRNA15	RNA gene finder		142				Le Rhun et al., 2016
		csRNA25			129				

*S. gordonii*	str. Challis substr. CH1	csRNA1	Non-coding RNA gene finder	NC	87	ND	ND	ND	Marx et al., 2010
		csRNA2-1			96				
		csRNA2-2			94				
		csRNA7			90				
		csRNA21			58				
		csRNA22			202				

*S. mutans*	UA159	csRNA23-1	Non-coding RNA gene finder	NC	79	ND	ND	ND	Marx et al., 2010
		csRNA23-2			81				
		csRNA24			152				

*S. gallolyticus*	UCN34	csRNA9	Non-coding RNA gene finder	NC	63	ND	ND	ND	Marx et al., 2010
		csRNA18			66				
		csRNA38			138				
		csRNA39			118				
		csRNA40-1			65				
		csRNA40-2			71				

*S. dysgalactiae* subsp. *Equisimilis*	GGS_124	csRNA14	Non-coding RNA gene finder	NC	68	ND	ND	ND	Marx et al., 2010
		csRNA15			141				
		csRNA16			127				
		csRNA17			117				

*S. equi* subsp. *equi*	4047	csRNA18	Non-coding RNA gene finder	NC	50	ND	ND	ND	Marx et al., 2010
		csRNA17			105				

*S. equi* subsp. *zooepidemicus*	MGCS10565	csRNA18	Non-coding RNA gene finder	NC	67	ND	ND	ND	Marx et al., 2010
		csRNA19			105				
		csRNA20			108				

*S. suis*	05ZYH33	csRNA26	Non-coding RNA gene finder	NC	172	ND	ND	ND	Marx et al., 2010
		csRNA27			73				
		csRNA28			58				

*S. uberis*	0140J	csRNA29	Non-coding RNA gene finder	NC	84	ND	ND	ND	Marx et al., 2010
		csRNA30			83				
		csRNA31			67				
		csRNA32			140				

*S. thermophilus*	St0 plasmid pSt0	csRNA9	Non-coding RNA gene finder	NC	60	ND	ND	ND	Marx et al., 2010

*S. thermophilus*	CNRZ1066	csRNA33	Non-coding RNA gene finder	NC	66	ND	ND	ND	Marx et al., 2010
		csRNA34			85				
		csRNA35			64				
		csRNA36			97				
		csRNA37			127				

*S. lutetiensis*	033	csRNA9	BLAST analysis	NC	63	ND	ND	ND	Denapaite et al., 2016
		csRNA18			66				
		csRNA38			137				
		csRNA39			119				

*S. infantis*	GTC849	csRNA2	BLAST analysis	NC	98	ND	ND	ND	Denapaite et al., 2016
		csRNA3			100				
		csRNA4			93				
		csRNA6			200				

*NC, Not confirmed; ND, Not determined.*

Except for *S. pneumoniae* and *S. sanguinis*, few studies regarding the role and targets of these csRNAs in other streptococci were conducted, although the importance of RNAs is highlighted.

## Discussion

The aim of this review is to carry out an inventory of the sRNAs regulated by the two-component regulatory system CiaRH present in streptococci. CiaRH TCS is conserved in all streptococci and controls many cellular processes including natural competence, virulence, and resistance to the immune system (Dagkessamanskaia et al., 2004; Sebert et al., 2005; Quach et al., 2009; Li et al., 2011). The csRNAs increase the regulatory networks of CiaR, which already directly controls more than 20 other genes (Halfmann et al., 2007). Promoters that drive the expression of the five csRNAs of *S. pneumoniae* are strongest in the CiaR regulon (Halfmann et al., 2007). The high proportion of sRNAs compared with other genes controlled by CiaRH indicates the importance of these csRNAs in bacterial regulation. Although csRNAs are predicted in various *Streptococcus* species and their importance highlighted, for most of them, no role or target has been identified until now. So far, only the csRNAs of *S. pneumoniae* and *S. sanguinis* have been investigated (Schnorpfeil et al., 2013; Laux et al., 2015; Ota et al., 2017). The study of csRNAs in those species has allowed the identification of different metabolic pathways in which csRNAs may be involved. Indeed, *S. pneumoniae* harbors five csRNAs, all implicated in competence development and thus, probably in horizontal transfer (Halfmann et al., 2007; Tsui et al., 2010). Moreover, two *S. pneumoniae* csRNAs (csRNA4 and csRNA5) seem to control bacterial autolysis (Halfmann et al., 2007). The involvement of csRNA5 in lung infection as well shows that each csRNA may be involved in different regulatory pathways (Mann et al., 2012). In this case, csRNA5 is on the one hand involved in competence development and on the other hand in virulence.

The investigation of *S. pneumoniae* csRNAs targets also allowed identifying different regulation pathways. According to the competence regulation previously mentioned, one target (*ComC*), encoding the competence-stimulating peptide precursor (CSP) was identified. This identification adds a proof concerning the involvement of *S. pneumoniae* csRNAs in horizontal transmission pathways. The Spr0159 target is most likely a transcriptional regulator: this suggests the involvement of the csRNAs in complex regulatory networks. Other identified targets (*spr0081, spr0371, spr0551*, and *spr1097*), encoding membrane spanning, belonging to different transporter families, indicate the possible involvement of csRNAs in stress resistance (Schnorpfeil et al., 2013). The four csRNAs identified in *S. agalactiae* NEM316 strain (*srn015*, *srn024*, *srn070*, and *srn085*) are overexpressed at low pH (5.2), suggesting their role in acid stress resistance (Rosinski-Chupin et al., 2015). Thus, the possible implication of csRNAs in stress tolerance in *S. pneumoniae* and *S. agalactiae* reveals a new regulation pathway in which csRNAs may play a role. In *S. sanguinis*, csRNAs are involved in host colonization by biofilm formation (Ota et al., 2017). This regulation of colonization by csRNAs has not yet been observed in other streptococcal species. Analysis of *S. pneumoniae, S. agalactiae*, and *S. sanguinis* csRNAs demonstrates that they are involved in a wide range of regulatory pathways. Indeed, the colonization, the virulence, the horizontal transfer, and maybe the resistance to environmental stress is affected by csRNAs. The various regulatory pathways in which csRNAs are involved can be explained by the diversity of csRNAs in each species and between streptococcus species. Moreover, as observed in *S. pneumoniae*, one csRNA can be involved in different regulatory pathways, thus increasing the complexity of regulatory networks.

The diversity of csRNAs between streptococcus species is remarkable (Table 1 and Supplementary Table 1). However, some species contain csRNAs from other species (*S. infantis* harbors csRNAs of *S. oralis* Uo5 and *S. lutetiensis* harbors the *S. gallolyticus* UCN34 csRNAs) (Denapaite et al., 2016). Moreover, *S. oralis* strains contain duplicated csRNAs genes. A genetic island of four genes is present between them but absent in strains without csRNAs gene duplication. Furthermore, this genetic island is integrated in *S. infantis* DD18 between two csRNAs (Denapaite et al., 2016). These data suggest that csRNAs are not only involved in gene regulation but may also contribute to horizontal gene transfers improving bacterial adaptation.

**FIGURE 2:**
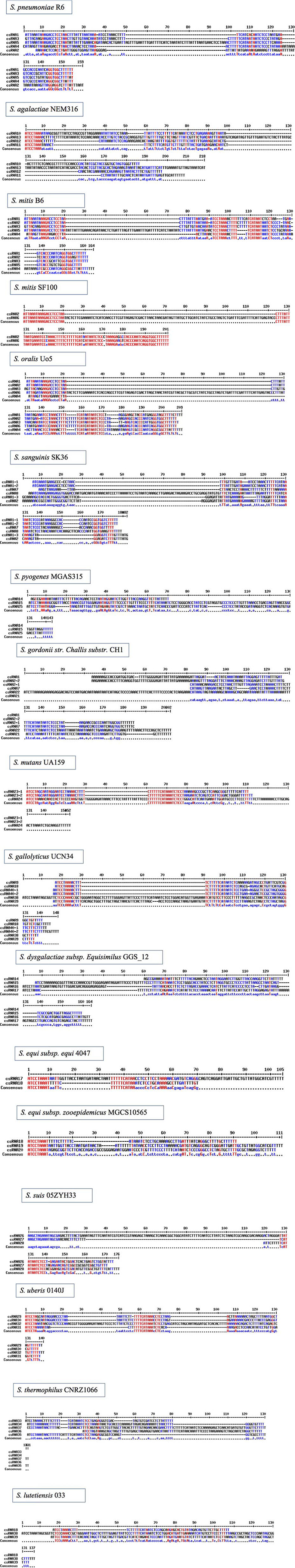
Alignments of csRNAs sequences by species by MultAlin. Nucleotides in red correspond to highly conserved sequences. Nucleotides in blue correspond to conserved sequences. Nucleotides in black correspond to non-conserved sequences.

In some species, csRNAs display a high degree of similarity to each other (Figure 2). This similarity is observed more particularly in regions complementary to RBSs and AUG start codons, suggesting that csRNAs bind to mRNA target and inhibit translational initiation. This would be fully consistent with the regulatory mechanism most commonly associated with sRNAs (Gottesman and Storz, 2011; Storz et al., 2011).

Other bacterial regulators also control multiple sRNA genes. For example, LuxO of *Vibrio harveyi* controls the expression of five sRNA genes (Tu and Bassler, 2007). The presence of regulators involved in sRNA regulation in various species suggests the importance of these sRNAs in bacterial adaptation and, beyond that, in bacterial survival.

Virulence and resistance to antibiotics and to the immune system mediated by CiaRH are possibly carried out through csRNAs. The discovery of Cbf1 protein that stabilizes all csRNAs in *S. pneumoniae* provides additional proof of the importance of csRNAs (Hör et al., 2020). In conclusion, understanding the csRNA-dependent regulatory network may contribute to the development of strategies against bacterial infections by targeting these sRNAs (Warner et al., 2018).

## Author Contributions

NJ and M-FL wrote the manuscript. Both authors contributed to the article and approved the submitted version.

## Conflict of Interest

The authors declare that the research was conducted in the absence of any commercial or financial relationships that could be construed as a potential conflict of interest.

## Supplementary Material

Supplementary Table 1 *Streptococcus* csRNAs sequences.
